# Genomic Characterization of Group C Orthobunyavirus Reference Strains and Recent South American Clinical Isolates

**DOI:** 10.1371/journal.pone.0092114

**Published:** 2014-03-14

**Authors:** Jun Hang, Brett M. Forshey, Yu Yang, Víctor Fiestas Solórzano, Robert A. Kuschner, Eric S. Halsey, Richard G. Jarman, Tadeusz J. Kochel

**Affiliations:** 1 Viral Diseases Branch, Walter Reed Army Institute of Research, Silver Spring, Maryland, United States of America; 2 U.S. Naval Medical Research Unit No. 6, Iquitos and Lima, Peru; 3 Centro Nacional de Salud Publica, Instituto Nacional de Salud, Lima, Peru; 4 U.S. Naval Medical Research Center, Silver Spring, Maryland, United States of America; The University of Texas Medical Branch, United States of America

## Abstract

Group C orthobunyaviruses (family *Bunyaviridae*, genus *Orthobunyavirus*), discovered in the 1950s, are vector-borne human pathogens in the Americas. Currently there is a gap in genomic information for group C viruses. In this study, we obtained complete coding region sequences of reference strains of Caraparu (CARV), Oriboca (ORIV), Marituba (MTBV) and Madrid (MADV) viruses, and five clinical isolates from Peru and Bolivia, using an unbiased *de novo* approach consisting of random reverse transcription, random anchored PCR amplification, and high throughput pyrosequencing. The small, medium, and large segments encode for a 235 amino acid nucleocapsid protein, an approximately 1430 amino acid surface glycoprotein polyprotein precursor, and a 2248 amino acid RNA-dependent RNA polymerase, respectively. Additionally, the S segment encodes for an 83 amino acid non-structural protein, although this protein is truncated or silenced in some isolates. Phylogenetically, three clinical isolates clustered with CARV, one clustered with MTBV, and one isolate appeared to be a reassortant or a genetic drift resulted from the high variability of the medium segment which was also seen in a few other orthobunyaviruses. These data represent the first complete coding region sequences for this serocomplex of pathogenic orthobunyaviruses. The genome-wide phylogeny of reference strains is consistent with the antigenic properties of the viruses reported in the original serological studies conducted in the 1960s. Comparative analysis of conserved protein regions across group C virus strains and the other orthobunyavirus groups revealed that these group C viruses contain characteristic domains of potential structural and functional significance. Our results provide the basis for the developments of diagnostics, further genetic analyses, and future epidemiologic studies of group C viruses.

## Introduction

The genus *Orthobunyavirus* (family *Bunyaviridae*) encompasses great genetic and antigenic diversity, with approximately 50 classified viral species belonging to a variety of serogroups, including Bunyamwera, California, group C, and Simbu [1]. Orthobunyaviruses are enveloped viruses that contain a tri-segmented negative-sense RNA genome, which encodes a large RNA-dependent RNA polymerase (L protein or RdRP) on the large (L) segment, a polyprotein precursor that comprises two envelope glycoproteins (Gc and Gn) and a non-structural protein (NSm) on the medium (M) segment, and a nucleocapsid protein (NP or N protein) and a non-structural protein (NSs) on the small (S) segment [2], [3]. Most orthobunyaviruses are transmitted by arthropods, and many are associated with human diseases, including several reported emerging infectious diseases [4], [5].

Group C viruses were named based on their serological characteristics distinctive from the historical group A (alphaviruses of the family *Togaviridae*) and group B (flaviviruses of the family *Flaviviridae*) arboviruses [6]. Since the first identification of group C viruses in Brazil in the 1950s, including Marituba virus (MTBV, strain BeAn15) and Oriboca virus (ORIV, strain BeAn17), a number of group C viruses have been isolated from humans, monkeys, other animals, and arthropods in all major tropical and subtropical regions in the Americas [7]–[11]. In humans, group C virus infections can produce fever with symptoms that are difficult to distinguish from dengue viral infection [12]. Virus classification is traditionally based on systematic serological assays, which are limited by the availability of specific and high quality antisera. Four major serological complexes within group C were established based on isolates collected during the 1950s and 1960s, which are represented by the species *Caraparu virus* (CARV), *Madrid virus* (MADV), *Marituba virus* (MTBV), and *Oriboca virus* (ORIV) in the International Committee on Taxonomy of Viruses (ICTV) database [1]. Despite their association with human illness, there is a paucity of genetic data for group C viruses. For example, there are no whole genome sequences or complete coding region sequences of all three segments for any group C virus publically available in GenBank database. The molecular studies that have been conducted were based on S segment sequences and/or partial sequences of L and M segments [9], [13], [14], and are therefore insufficient for accurate phylogenetic characterization.

To address the scarcity of genome sequences of group C viruses, the four reference strains which are listed by the ICTV (http://ictvonline.org/) to represent the four established serological complexes within the group C were subjected to whole genome sequencing using unbiased random amplification and next-generation sequencing. In addition, five group C virus isolates collected from participants in an acute febrile illness surveillance study in Peru and Bolivia between 2003 and 2008 [11] were sequenced. Phylogenetic relatedness among the references, the recent clinical isolates, and more distantly related orthobunyaviruses was established and comparative analysis of complete coding region sequences was conducted.

## Materials and Methods

### Viruses, viral culture, RNA purification

Group C virus reference strains (Table 1) were obtained from the American Type Culture Collection (ATCC). Clinical specimens (Table 1) were isolated from acute-phase sera of patients identified through a clinic-based febrile surveillance program run jointly by the US Naval Medical Research Unit No. 6 and the Peruvian Ministry of Health [11]. The clinical specimens used in this study were obtained under the terms of a human use protocol (NMRCD.2000.0006). Written, informed consent was obtained from the participants or from a parent or legal guardian. The human use protocol and the consent procedure were approved by the Naval Medical Research Center Institutional Review Board (Bethesda, MD, USA) in compliance with all U.S. federal regulations governing the protection of human subjects. Clinical isolates were provisionally classified as group C viruses based on reactivity with CARV and Murutucu virus (MURV, a member of the MTBV serocomplex) antibodies in an immunofluorescence assay, as previously described by Forshey et al [11].

**Table 1:** Group C virus reference strains and recent South American isolates used in the study.

Virus Identity	Source institution, Catalog number	Year	Isolation location	Tissue origin	GenBank Accession Numbers for sequences from this study
Marituba (MTBV) BeAn15	ATCC, VR-308	1954	Belem, Brazil	*Cebus apella* serum	KF254770 - KF254772
Oriboca (ORIV) BeAn17	ATCC, VR-310	1954	Belem, Brazil	*Cebus apella* serum	KF254773 - KF254775
Caraparu (CARV) BeAn3994	ATCC, VR-307	1956	Belem, Brazil	*Cebus apella* serum	KF254776 - KF254778
Madrid (MADV) BT 4075	ATCC, VR-385	1961	Bocas del Toro, Panama	Serum from a 36-year-old male	KF254779 - KF254781
IQE7620	NAMRU6	2008	Iquitos, Peru	Serum from a 14-year-old female	JN157805 KF254782 KF254783
FVB0426	NAMRU6	2008	Cochabamba, Bolivia	Serum from a 24-year-old female	KF254784 - KF254786
IQD5973	NAMRU6	2003	Iquitos, Peru	Serum from a 17-year-old female	KF254787 - KF254789
FSL2923	NAMRU6	2006	Yurimaguas, Peru	Serum from a 59-year-old male	KF254790 - KF254792
FMD0783	NAMRU6	2006	Puerto Maldonado, Peru	Serum from a 21-year-old male	KF254793 - KF254795

ATCC, the American Type Culture Collection.

All virus strains were propagated in Vero (African green monkey kidney) cells and harvested upon appearance of cytopathic effect (CPE). Viral culture supernatants were clarified by centrifugation and precipitated by the addition of 1.4 g polyethylene glycol (PEG) 8000 and 0.47 g NaCl to each 10 ml of clear supernatant and refrigerated overnight. After centrifugation at 3200×g for 30 min at 4°C, the pellets were resuspended with 0.5 ml of PBS containing magnesium and calcium and 0.5 μl of Benzonase nuclease (12.5 U/μl) (Sartorius Stedim, Germany) and incubated at 37°C for 30 min. After treatment, RNA was extracted using Trizol LS (Invitrogen).

### Random reverse transcription and amplification, high throughput pyrosequencing

RNA extracts were reverse transcribed and amplified using anchored random octamer oligos. Random amplicons were ligated to Roche GS RL adaptors and size selected to recover libraries of 250 bp or larger DNA molecules. Roche GS FLX Titanium or FLX+ system was used to sequence the libraries using the manufacturer recommended protocols with modifications. The procedure development and its application to *de novo* sequencing of a novel viral sequence was described in detail previously [15].

### Pyrosequencing data analysis, genome sequence assembly and analysis

Roche GS data analysis software GS De Novo Assembler version 2.5.3 was used in *de novo* assembly of the pyrosequencing data. GS Reference Mapper version 2.5.3 was used for reference mapping sequence assembly. Other bioinformatics tools and the applications in the study included MEGA 5.2.1 for phylogenetic analysis [16], Geneious Pro version 5.6.4 (Biomatters Ltd, Auckland, New Zealand) for visualization of sequence assembly results, sequence editing, alignment and phylogenetic analysis, NCBI discontiguous Megablast (http://blast.ncbi.nlm.nih.gov/Blast.cgi) [17] for alignment of sequences with low identity, NCBI ORF Finder (http://www.ncbi.nlm.nih.gov/gorf/orfig.cgi) for finding open reading frames and identification of conserved regions by searching against the protein sequence database, and Sequin software for the annotation and compiling of GenBank format files. Prior to phylogenetic analyses, nucleotide or amino acid sequences were aligned using program MUSCLE [18], edited to trim sequences from both termini that could not be reliably aligned, then realigned. Phylogenetic distance trees were subsequently constructed by using the Neighbor-Joining (NJ) method and the Tamura-Nei model with the use of the Maximum Composite Likelihood model to verify the analytical results. Deduced protein sequences were submitted to PROMALS web server (http://prodata.swmed.edu/promals/) for multiple protein sequence alignments, secondary structure prediction and determination of conservation indices for the amino acid residues using AL2CO program [19], [20].

## Results

### Genome segments and encoded proteins

We sequenced the four group C virus reference strains and five recent isolates (Table 1) by using an unbiased approach. High sequence coverage depth (67-fold or greater in average sequence alignment coverage) was obtained for each virus (Table 2). Sequences for genome segments obtained using random amplification and pyrosequencing comprise complete coding sequences and partial terminal untranslated sequences. The deduced amino acid sequences from the nucleotide sequences acquired in this study have similar amino acid lengths with other orthobunyavirus complete coding regions (Table 2), except for the Brazoran virus which has a 1.7 kb S segment encoding putative 172 amino acid NSs and 442 amino acid N protein [21]. The amino acid similarities are consistent with the established taxonomic relationships [1]; specifically, group C virus isolates shared less than 55%, 35%, and 45% pairwise amino acid identity with members of other orthobunyavirus serogroups for the L, M, and S segments, respectively (Supplementary Table S1, white areas). Pairwise amino acid identities within the group C, based on sequences from this study, ranged from 79.4% to 99.4% for the L segment, 65.2% to 98.7% for the M segment, and 71.5% to 98.3% for the S segment (Table S1, shaded areas). All segments of recent clinical isolates showed high sequence identity to segments of one or more of the reference viruses, with the exception of the M segment of isolate FSL2923 (Table S1). FVB0426, FMD0783, and IQD5973 showed greater than 96% pairwise amino acid identities with CARV for all three segments. IQE7620 segment sequences were most similar to MTBV, with the amino acid identities of 92.8%, 90.5% and 97.5% for L, M and S respectively. L and S segments for FSL2923 were highly similar with CARV, while its M segment was almost equally divergent from CARV and MADV, with nucleotide (amino acid) identities of 75.3% (83.2%) and 76.0% (84.8%), respectively (Table S1).

**Table 2:** Group C virus genome sequences and deduced amino acid sequences.

		L segment		M segment		S segment		
Virus	Average Sequencing Depth	Genome(nt)	RdRP (aa)	Genome (nt)	Gn/NSm/Gc (aa)	Genome(nt)	NP (aa)	NSs (aa)
Bunyawera virus (NC_001925-7)	NA	6875	2238	4458	1433	961	233	101
La Crosse virus (NC_004108-10)	NA	6980	2263	4527	1441	984	235	92
Simbu virus (NC_018476-8)	NA	6895	2253	4417	1409	860	233	91
Oropouche virus (NC_005775-7)	NA	6846	2250	4385	1420	754	231	91
Marituba (MTBV) BeAn15	273	6894	2248	4594	1435	1111	235	62*
Oriboca (ORIV) BeAn17	2165	6895	2248	4332	1429	1032	235	62*
Caraparu (CARV) BeAn3994	396	6855	2248	4356	1430	1048	235	83
Madrid (MADV) BT4075	825	6872	2248	4642	1429	1090	235	83
IQE7620	210	6936	2248	4538	1431	1008	235	62*
FVB0426	127	6850	2248	4352	1430	1049	235	83
IQD5973	67	6794	2248	4392	1430	1039	235	83
FSL2923	161	6854	2248	4371	1429	966	235	83
FMD0783	465	6849	2248	4349	1430	1028	235	83

Sequences for genome segments obtained in this study include complete coding sequences and partial terminal untranslated sequences, except IQE7620 L segment sequence is complete. Four representative orthobunyaviruses of other serogroups are shown for comparison. * Three NSs open reading frames are predicted to be N-terminus truncated which may not express.

### Phylogenetic relationship based on whole genome sequences

**Figure 1:**
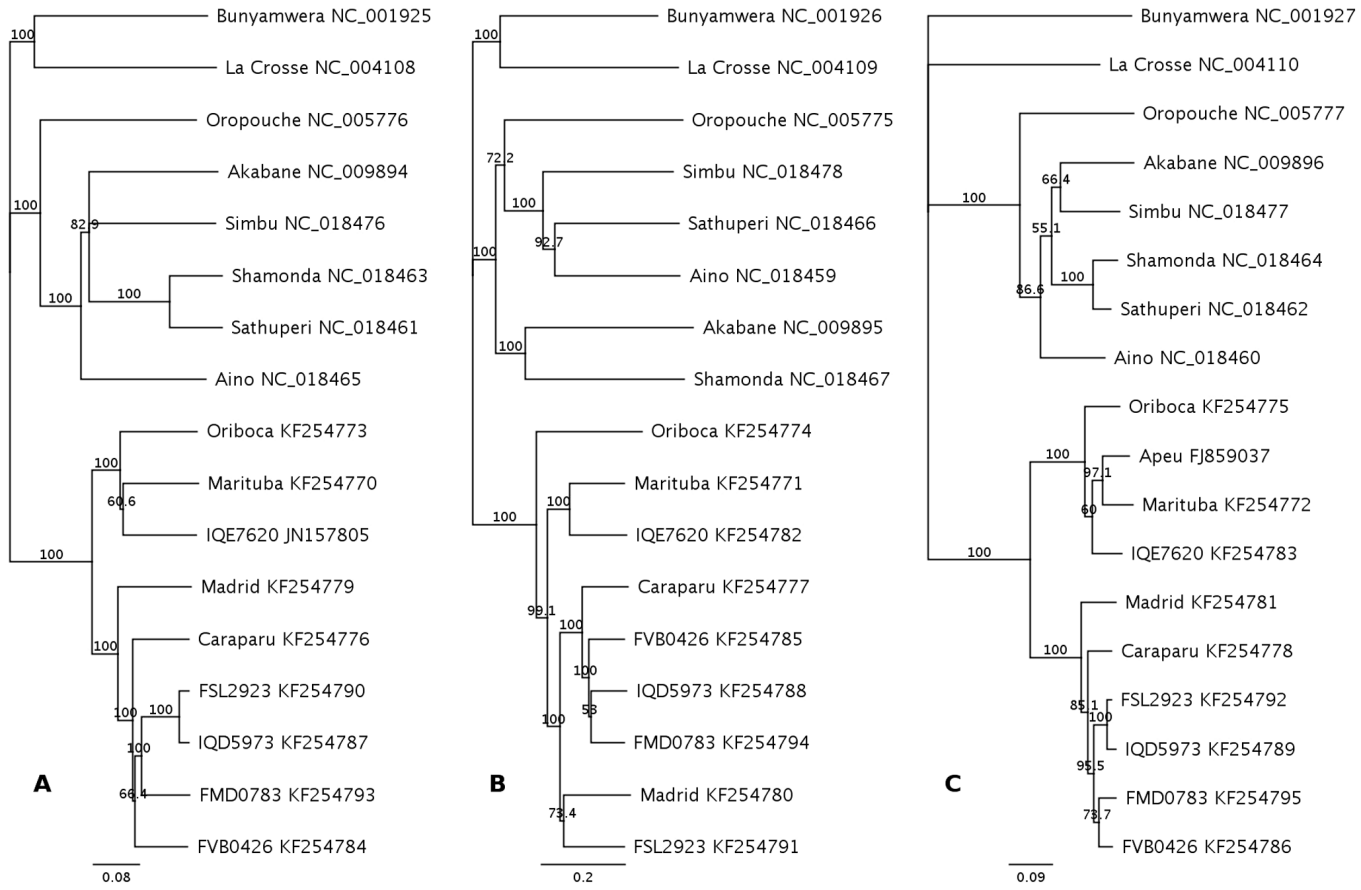
Genome-wide phylogenetic analyses based on nucleotide sequences for orthobunyaviruses. Complete coding sequences for nine group C viruses from this study and GenBank reference genome sequences (Refseq) for eight orthobunyavirus were aligned with MUSCLE, trimmed to remove the unaligned termini, and used in construction of the phylogenetic trees by the Neighbor-Joining method and Tamura-Nei model for (**A**) L genome segment, (**B**) M genome segment and (**C**) S genome segment. Complete coding sequence for S segment of Apeu virus BeAn 848 was also included. The scale bars indicate nucleotide substitutions per site. Reliability of the branching patterns was assessed by bootstrap method with the use of 1000 replicates (shown at nodes). Virus names, strain identities and GenBank accession numbers are shown.

To determine the evolutionary relationships among orthobunyaviruses of other serogroups, phylogenetic trees based on complete coding sequences were constructed for the L, M and S segments (Figure 1). Sequences for all group C viruses clustered together in a clade distinctive from other orthobunyavirus groups. For all three trees, MADV and CARV were on neighboring branches, distinct from MTBV and ORIV. Overall, MTBV and ORIV had a close phylogenetic relationship, in particular for their S and L segments. M segment for MTBV was slightly closer to MADV/CARV than to ORIV. FVB0426, FMD0783, and IQD5973 were all closely related to CARV, while IQE7620 was more related to MTBV. For FSL2923, the L and S genome segments were both phylogenetically more closely related to CARV, but the M segment was more closely related to MADV.

### Protein sequence conservation analysis and identification of variable regions

**Figure 2:**
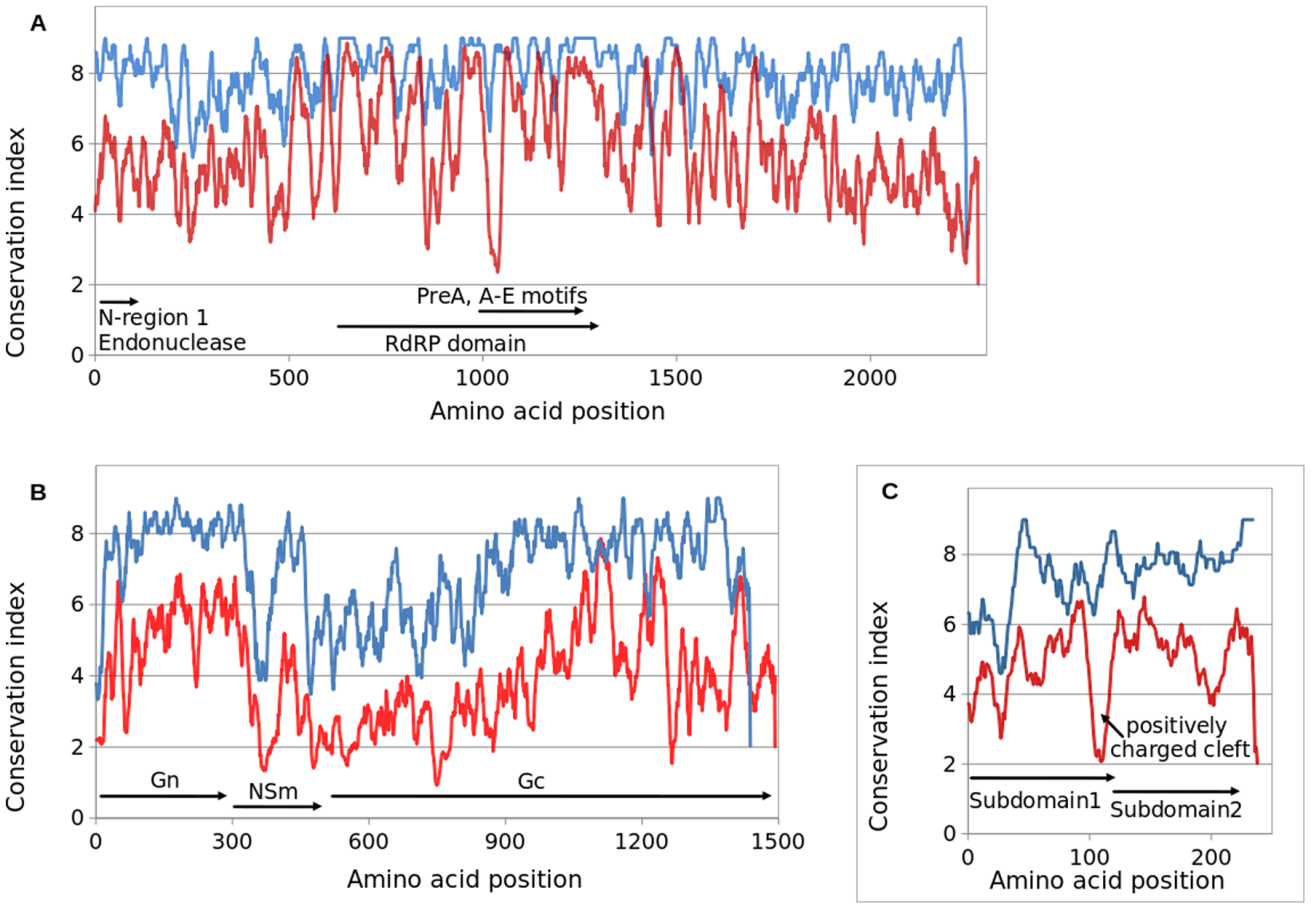
Analyses of protein conservation for (A) L protein, (B) M polyprotein precursor and (C) N protein. Protein sequences were aligned by the PROMALS server. The conservation index numbers (0–9 for the lowest to the highest) were averaged with a window size of 15 amino acids and then plotted to show amino acid conservation and variability. *Blue curves*, all nine group C viruses sequenced in this study. *Red curves*, the group C viruses and orthobunyaviruses of other serogroups (reference sequences in GenBank). Relative positions for Gn, NSm and Gc proteins on M segment and structural domains on L and S segment were indicated with the arrowed bars.

Conserved protein regions across group C virus strains and the other orthobunyavirus groups were explored to show whether these group C viruses contain characteristic domains of potential structural and functional significance (Figure 2). Overall, on genus level, the L protein is highly conserved, although amino acid sequences at both termini are slightly less conserved than the central region. The predicted group C virus L protein RdRP has a strict length of 2248 amino acids (Table 2). The putative RdRP catalytic domain is located within amino acid residues 597–1330 (group C virus numbering) [22]. The region contains multiple extremely conserved motifs including the designated premotif A, motif A-E, and all 16 residues strictly conserved across the family *Bunyaviridae* as well as in some other negative-stranded RNA viruses [23]. The previously identified N-terminal conserved region 1 (15 to 143) and region 2 (634 to 779), the intermediate variable linker region, all key region 1 residues (R36, H37, F40, P81, D82, D95 and K97), and key region 2 residues (R657 and Y658) [23] were also found in all group C virus L proteins. The predicted consensus secondary structure for the N-terminus of group C virus L proteins has an arrangement of α1-3β1β2α4β3β4α5β5α6α7, which is nearly identical to that from the crystal structure for LC183, N-terminal 183 amino acid fragment of *La Crosse virus* (LACV; genus *Orthobunyavirus*) [24], except that a short motif, IVVDIN / ITLNVT for LACV / CARV was predicted to form β5 but not revealed in the LC183 crystal structure. Based on the high structural similarity, L protein N-terminal domain of group C viruses likely also has endonuclease activity similar to PA polymerase N-terminal domain of influenza [24].

We compared group C virus amino acid residues in the N protein with residues previously reported to be conserved across other orthobunyavirus serogroups. Over sixty key functional residues were proposed to be crucial for the hydrophobic core structure, the RNA binding sites and NP-NP oligomerization interface, based on mutational analysis and determined crystal structures of orthobunyavirus NPs [25], [26]. These residues were considered absolutely genus-conserved and, as expected, almost all of them were found identical (46/63) or chemically similar (11/63) in group C virus NP sequences. Notably, polymorphic sites were found at the following positions (6/63) in some group C viruses: P84 to S/T, H93Y, E150 to I/V, T155 to D/E, P160S, and A191P (LACV numbering, Table 3). Additional amino acid sites in NP previously reported to be absolutely conserved across the orthobunyavirus genus [25], [27], but varied in our group C viruses include F158M, V168A, and M173I. Many of these residues are located in regions outside the core structural and functional domains. This polymorphic variance also presents in other recent orthobunyavirus sequences with variation rates lower than in group C viruses (Table 3).

**Table 3:** Amino acid differences for the key functional residues of nucleoprotein (NP).

La Cross	MTBV	ORIV	MADV	CARV	Other group C viruses	Other orthobunyaviruses
Pro84	**Thr87**	**Thr87**	Pro87	Pro87	FMD0783/FVB0426 **Ser87**; Apeu **Thr87**	I612045 **Thr106**; Oyo **Thr109**; Wyeomyia **Asp84**
His93	His96	His96	**Tyr96**	**Tyr96**	FSL2923/IQD5973/FMD0783 /FVB0426 **Tyr 96**	I612045 **Asn106**; Oyo **Asn118**
Glu150	Glu150	Glu150	**Ile150**	**Val150**	FSL2923/IQD5973/FMD0783 /FVB0426 **Ile150**	
Thr155	**Glu155**	**Glu155**	**Glu155**	**Glu155**	FSL2923/IQD5973/FMD0783 /FVB0426 **Glu155**; IEQ7620 **Asp155**; Apeu **Glu155**	I612045 **Glu174**; Oyo **Glu177**; Leanyer **Asp156**
Pro160	Pro160	Pro160	**Ser160**	**Ser160**	FSL2923/IQD5973/FMD0783 /FVB0426 **Ser160**	
Ala191	**Pro191**	**Pro191**	**Pro191**	**Pro191**	FSL2923/IQD5973/FMD0783 /FVB0426/IEQ7620 **Pro191**; Apeu **Pro191**	Oyo **Pro214**

Amino acid residues corresponding to the key residues for La Cross virus NP but with significant differences (shown in bold) were listed for the nine group C viruses and other orthobunyavirus species, which include NPs for Apeu (ACZ65466), I612045 virus (AED98378), Oyo (AEE01389), Leanyer (AEA02984) and Wyeomyia (AEZ35275). Other orthobunyavirus NPs that were compared but have no differences at these residue positions include Bunyamwera, Oropouche, Sathuperi, Shamonda, Akabane, Simbu, Aino, Guaroa (CAA51847), Manzanilla (AFI24665), and Schmallenberg (CCF55031) viruses.

The predicted NSs protein for most group C viruses is 83 amino acids in sequence length, shorter than most other orthobunyaviruses NSs proteins, which generally include 91–101 amino acids. Pairwise amino acid identity between species ranges from 96.8% (MADV vs CARV) to 45.2% (CARV vs ORIV) within group C and as low as 22.6% (CARV/MADV vs Bunyawera) when compared with other orthobunyaviruses [2]. The start codon ATG (AUG) is mutated to TTG (UUG) in the homologous NSs coding regions for MTBV BeAn15, ORIV BeAn17, and isolate IQE7620. Since TTG is rarely used in eukaryotes to initiate translation [28], the NSs protein translations for these isolates might be fully silenced or truncated by 21 amino acids if translation is initiated at next in-frame ATG. The absence or truncation of NSs ORF was shown or speculated in other orthobunyaviruses [29], [30]. The actual alteration of NSs translation and the functional consequences are yet to be elucidated.

Consistent with other orthobunyaviruses, the M segment of group C viruses encodes a protein of approximately 1430 amino acids (Table 2). Proteins encoded on M segments (Gn, NSm, and Gc) have variable level of conservation. Gn is highly conserved among group C viruses except for at the extreme N-terminus. NSm is very poorly conserved at the N- and C-termini, but highly conserved in between. The Gc gene products are poorly conserved in the N-terminal half of the protein, but more highly conserved in the C-terminal half (Figure 2), which is in line with the functional delineation study which suggested that N-terminal half of Gc is not essential for its structure and function in cell culture [31]. In addition, characteristic amino acid residues and motifs conserved across orthobunyaviruses were found to be well preserved in group C viruses. These include the potential N-linked glycosylation sites, trypsine cleavage sites, a number of cysteine residues, etc. [29], [32], [33]. M segment polyprotein of MTBV has 72 cysteines in total, 58 of which are strictly conserved among known orthobunyaviruses. All except 2 of the remaining 14 cysteines are conserved in all group C viruses.

## Discussion

Group C virus reference strains used in this genomic study were identified and established as references for each of the four major serological complexes of group C decades ago [6]. However, further categorization has been hampered by a paucity of genetic data for these reference strains. Our data provide a basis for comparing genetic and serological relationships among group C viruses. The serological relationships among the references were defined by hemagglutination-inhibition (HI) and neutralization tests (NT), which are dependent on antigenic properties of the surface glycoproteins (Gn and Gc), and complement fixation (CF) tests, which are dependent on N protein antigenic characteristics [1], [6]. MADV and CARV display some cross-reaction in both HI and CF assays. In contrast, MADV and CARV are very poorly cross-reactive with MTBV or ORIV in HI and CF tests [8]. MTBV weakly cross-reacted with both ORIV and CARV in HI and NT, but only cross-reacted with ORIV in CF [6]. We found these serological characterizations [34] consistent with the gene segment-specific phylogenetic relationships. For instance, ORIV and MTBV are on a same branch of the phylogenetic tree for S segment, which encodes N protein that attributes to CF antigenic activity but not for the M segment, which is associated with HI results.

The genome-wide phylogenetic analysis provides an important reference for primer design for diagnostics and additional molecular evolutionary analysis. The sequence alignment and conservation analysis revealed sequence and structural variations within the genome. This knowledge is informative for choosing a genome region(s) suitable for molecular assay development and phylogenetic analysis. These data are necessary for understanding the true extent of viral diversity and for understanding the epidemiology and epizoology of these viruses. In general, regions of the S and M segments are better suited than the most conserved L segment for discriminating diverse isolates unless the whole or a large portion of the L sequence is used. In particular, the 5′-terminal nucleotide sequence for glycoprotein Gc is highly variable, and is therefore well-suited for distinguishing closely related strains; while Gc sequences close to 3′- nucleotide terminus are highly conserved within the serogroup and moderately variable among the different serogroups, thus adequate for comparing isolates with high divergence or isolates of different species.

Current rules for species demarcation for orthobunyaviruses are not clear. Orthobunyaviruses are widely present throughout the world and enormously diverse, yet genetic and biochemical data is limited. Current guidelines from the ICTV for species definition are based on serological criteria (cross - neutralization and cross - hemagglutination inhibition tests), low likelihood of reassortment between species, and N protein amino acid divergence of more than 10% [1]. This pattern seems not supported by group C virus profiles found in this study. It does not apply well even to the reference strains which were proven antigenically distinct from each other – amino acid sequence identities for NP are 96.2% between ORIV and MTBV and 94.5% between CARV and MADV. This study suggests the need for future revision of the speciation criteria. Based on L segment sequence and comparison with existing data in GenBank, IQE7620 was provisionally named as Zungarococha virus (ZUNV) because of the low nucleotide and amino acid sequence identity with other reported orthobunyaviruse sequences [15]. Further sequence analyses in this study suggests that ZUNV likely does not represent a novel species, but rather is a member of the MTBV serocomplex, because of its <10% amino acid divergence in the N protein, as well as in the RdRP and M polyprotein. Our study showed M segment sequence can distinguish group C from other serogroups and effectively differentiate the four references from each other with >15% amino acid divergence; moreover, the phylogenetic dendrogram is in agreement with the antigenic relatedness defined primarily by the surface glycoproteins encoded by M segment. Therefore, we suggest M sequences are taken into account together with L and S sequences as criteria of sequence similarity for classification into species.

A further complication of species demarcation is the potential for reassortment. We observed that S segment based phylogeny was always consistent with L segment phylogeny. In contrast, M segment phylogeny was discrepant from L and S phylogenies for three (MADV, MTBV, FSL2923) out of nine group C isolates. This phenomenon appears to be applicable to other orthobunyaviruses that were presumably ascribed to M segment reassortant (Figure 1B) [29], [33], [35], [36]. RdRP and NP are both components for the essential ribonucleoprotein complex, and functionally and structurally associated [37], which may restrict the viability of reassortants. M segment and the surface proteins are closely related to vector specificity, infectivity and escape from host immunity. Hypervariation of M sequences might allow for adaptation to a new vector or host. In this way, viruses from the same ancestor might become less alike in their M segments when exposed to distinctive immune selective pressures. As for the group C reference viruses, MADV/CARV and ORIV/MTBV might have respectively originated from two different ancestors and evolved into distinct species due to accumulated divergence on M sequence. Another example is the newly identified livestock pathogen Schmallenberg virus (SBV), which shares 98.7–99.1% (L), 89.7–90.2% (M) and 96.1–97.2% (S) amino acid identities to the Japanese Sathuperi viruses [35]. Moreover, in recent studies on sequence variability of SBV, M segment sequence was shown more variable than L and S, with a hypervariable region at the N-terminus of Gc protein [38], [39]. *In vitro* successive SBV passage on baby hamster kidney cell line BHK-21 led to marked accumulation of mutations concentrated on the M segment hypervariable region [39]. Further investigation in vectors and hosts will provide more definitive insights on the molecular and antigenic evolution of the orthobunyaviruses.

Group C virus sequences from this study are in good agreement with data from other studies, including nine sequences in GenBank [13], [23], with a remarkable exception for the 39 sequences (DQ188946 – DQ188984) by Nunes et al (see below) [14]. CARV BeAn3994 sequence determined here is nearly identical (99.78%) to the 5555 nucleotides 5′-terminal L segment sequence for CARV BeAn3994 (EF122411, nucleotide identity 5532/5544, RdRP amino acid identity 1829/1833) [23]. Similarly, a 299 nucleotide / 99 amino acid M segment fragment (AF499012) from Vinces virus, a member of the CARV serocomplex, is >95% identical at the amino acid level to CARV. Partial L (FL859039), partial M (FL859038) and complete S segment (FL859037) sequences from a clone of Apeu virus (APEUV, a member of the CARV serocomplex) strain BeAn848 [13] have amino acid identity of 73.9%, 92.9%, and 72.2%, respectively, with CARV; and 92.8%, 81.4%, and 97.9%, respectively, with MTBV (S segment phylogeny shown in Figure 1C). This sequence divergence pattern explains serological test results, in which CARV cross-reacted strongly with APEUV in HI tests but poorly in CF test, while APEUV BeAn848 cross-reacted strongly with MTBV in CF test [6], [34]. The results also suggest the evolutionary association of L and S segments, as described above, and the possibility that BeAn848 is a reassortant of MTBV and M segment of CARV.

The sequences by Nunes et al [14] were divergent from sequences derived in this and other studies, despite ostensibly originating from the same strains of group C viruses. For example, CARV strain BeAn3994 from our study shared only 35.0% S segment amino acid identity with the same CARV strain from the Nunes et al study. Similarly, de Brito Magalhes et al found that the S segment sequence of APEUV BeAn848 (FJ859037) has amino acid identity of only 35.0% and 33.3%, respectively, to sequences reported by Nunes et al for APEUV (strain BeAn848; DQ188952) and CARV (DQ188948), which were even lower than the identity (41.5%) with Bunyamwera (AF325122) [13]. As described in Forshey et al. (2014) [40], we were unable to amplify viral RNA using the primers published by Nunes et al, despite repeated attempts. Others have reported a similar inability to amplify group C orthobunyavirus RNA with the published primers (Lambert et al) [41]. Using our unbiased *de novo* sequencing approach, we generated full coding region sequences and could not identify clear complimentary sites for binding of the published primers. For these reasons, we have chosen to exclude the data from the Nunes *et al* study [14] from our analyses.

To date, group C viruses have not been associated with large scale outbreaks in humans, and the natural maintenance cycle is poorly understood. In Trinidad, all 16 group C virus isolates were collected from *Culex* (*Melanoconion*) spp. [42]. In an arbovirus surveillance study among mosquitoes in the Amazon Basin region of Peru, 164 viruses were isolated, among them 69 were orthobunyaviruses, 42 of which were group C viruses, predominantly carried by mosquitoes belonging to *Culex (Melanoconion)* spp. [10]. The frequent identification of group C viruses from mosquitoes in the area is consistent with our observation from a clinic-based febrile illness surveillance program, which demonstrated that orthobunyaviruses accounted for approximately 2.5% of all febrile cases, and 30 out of 54 orthobunyavirus isolates belonged to group C [11]. Infection by group C viruses causes a mild to severe illness. Since current surveillance studies are mainly focused on cases with clinical significance, the prevalence of the viral infections is likely underestimated and warrants further exploration. The sequence data presented here will facilitate surveillance investigations and ultimately a more comprehensive understanding of the genetic diversity, evolution mechanism, ecological niches and epidemic potential of the group C viruses.

Without thorough study of group C viruses, we cannot rule out the possibility that it may cause severe disease in humans or animals as Oropouche and Schmallenberg viruses have done [43], [44]. Group C viruses continue to circulate and cause diseases in humans throughout the Americas, indicating the need for continued surveillance and in-depth research on this group of diverse viruses. Future studies will focus on genomic and serological analysis of a broader panel of isolates from South America using primers designed based on the sequences reported here. Comparative genomic study of these related viruses, together with other orthobunyaviruses, will help clarify taxonomic relationships and delineate antigenic sites responsible for host specificity and adaptation. Additionally, sequences for terminal untranslated regions (UTRs) will also be pursued by using method such as 5′/3′ rapid amplification of cDNA ends (RACE) sequencing [21], not only to accomplish complete genome sequences, but also to investigate the specific roles of the conserved and variable UTR sequences in the virus structure and regulation of biological processes. Joining molecular and genomic approaches with serological tests, and including clinical samples from a broader geographic and temporal spectrum, will help us better understand the distribution of these viruses and the potential for broader emergence.

## Supporting Information

Table S1 **Percent sequence identities between orthobunyaviruses.** Complete coding sequences for nine group C viruses (this study) and eight orthobunyaviruses reference sequences (RefSeq of GenBank) were aligned with MUSCLE, trimmed to remove non-aligned terminal sequences and calculated for sequence identity percentages. Both nucleotide sequences (L, M and S segments) and correspondent protein sequences (L protein, M polyprotein and N protein) were analyzed with the results shown in the *upper-right* and *lower-left* of the tables, respectively. *Shaded* areas, results for comparison between viruses of same serogroup, i.e., Simbu group or group C viruses. Names of group C reference viruses are shown in bold.
(DOCX)
