# Supplementary material for: Genomic Characterization of Group C Orthobunyavirus Reference Strains and Recent South American Clinical Isolates
Source: PLoS One. 2014 Mar 14;9(3):e92114. doi: 10.1371/journal.pone.0092114 (PMC3954874; doi:10.1371/journal.pone.0092114)
Supplement: Table S1 — Percent sequence identities between orthobunyaviruses. Complete coding sequences for nine group C viruses (this study) and eight orthobunyaviruses reference sequences (RefSeq of GenBank) were aligned with MUSCLE, trimmed to remove non-aligned terminal sequences and calculated for sequence identity percentages. Both nucleotide sequences (L, M and S segments) and correspondent protein sequences (L protein, M polyprotein and N protein) were analyzed with the results shown in the upper-right and lower-left of the tables, respectively. Shaded areas, results for comparison between viruses of same serogroup, i.e., Simbu group or group C viruses. Names of group C reference viruses are shown in bold. (DOCX) [file pone.0092114.s001.docx]

**Table S1.** Percent sequence identities between orthobunyaviruses. Complete coding sequences for nine group C viruses (this study) and eight orthobunyaviruses reference sequences (RefSeq of GenBank) were aligned with MUSCLE, trimmed to remove non-aligned terminal sequences and calculated for sequence identity percentages. Both nucleotide sequences (L, M and S segments) and correspondent protein sequences (L protein, M polyprotein and N protein) were analyzed with the results shown in the *upper-right* and *lower-left* of the tables, respectively. *Shaded* areas, results for comparison between viruses of same serogroup, i.e., Simbu group or group C viruses. Names of group C reference viruses are shown in bold.
